# Alice in Wonderland Syndrome: Localising insights from right visual cortex stroke complicated by epilepsia partialis continua

**DOI:** 10.1016/j.ebr.2025.100745

**Published:** 2025-01-29

**Authors:** Gashirai K. Mbizvo, Viraj Bharambe, Brython Hywel, Shubhabrata Biswas, Andrew J. Larner

**Affiliations:** a Pharmacology and Therapeutics, Institute of Systems, Molecular and Integrative Biology, University of Liverpool, United Kingdom; b Liverpool Centre for Cardiovascular Science, University of Liverpool and Liverpool Heart & Chest Hospital, Liverpool, United Kingdom; c Neurology Department, The Walton Centre NHS Foundation Trust, Liverpool, United Kingdom; d Neurophysiology Department, The Walton Centre NHS Foundation Trust, Liverpool, United Kingdom; e Neuroradiology Department, The Walton Centre NHS Foundation Trust, Liverpool, United Kingdom; f Department of Brain Repair & Rehabilitation, Institute of Neurology, University College London, London, United Kingdom

**Keywords:** Syndrome of distorted perception, Status epilepticus, Myoclonus, Cerebral infarct, Cerebrovascular disease

## Abstract

The Alice in Wonderland syndrome comprises visual and/or somaesthetic distortions, most often migrainous in origin. We present a case of Alice in Wonderland syndrome in association with a right posterior/visual cortex infarction with subsequent seizures including focal motor status (epilepsia partialis continua), the latter a hitherto unreported association. These pathophysiological changes were of right hemisphere origin, suggesting Alice in Wonderland syndrome, if present, might have localising value for seizure focus in the right hemisphere.

## Highlights

• Alice in Wonderland syndrome (AIWS) consists of visual or somaesthetic distortions.
• These have enough perceptual stimulus to account for them, unlike hallucinations.
• AIWS is commonly migrainous, but can also be due to stroke or epilepsy.
• Epileptic AIWS mainly associates with right hemisphere pathology, hence localising.
• Pathophysiology of AIWS may be a failure of visual and somatosensory integration.

## 1 Introduction

Of the various neurological phenomena which are described in the works of Lewis Carroll, the pseudonym of Charles Lutwidge Dodgson [1], the “Alice in Wonderland syndrome” (AIWS) has perhaps gained the most traction in clinical practice [2], [3], [4], [5]. The name was originally coined to describe the altered perceptions of body image [6], such as the micro- or macrosomatognosia graphically pictured in the original publication of *Alice’s Adventures in Wonderland* in 1865 by Carroll’s illustrator Sir John Tenniel [7].

As currently understood, a large variety of perceptual distortions, both visual (metamorphopsia) and somaesthetic, may occur in AIWS. Classification is dependent on the nature of the distortions: A = somaesthetic; B = visual; C = both [5]. Diagnostic criteria have been proposed [2]. In adults, the most prevalent cause is migraine, although other conditions have also been reported to give rise to the phenomena, including infective, cerebrovascular, epileptic, drug-related, psychiatric and neurodegenerative disorders. We present a further case with an unusual association, possibly causal.

## 2 Case

In-patient neurological consultation was requested for a 69-year-old man with “visual hallucinations”.

For some weeks, he had had intermittent distortions of his vision. In these, people might appear to him as shorter or taller than they actually were, or the room in which he was located might appear to elongate or become shorter. In addition, he sometimes felt that parts of his own body, such as his head or his left knee, had become larger. He also reported some difficulties with colour perception, saying that he could not see things as well if they were not brightly coloured. He felt that the disturbances were more noticeable in his left eye. He was aware that these distortions were not real. They were not associated with any headache, photophobia, nausea, vomiting, or paraesthesias. There was no history compatible with visual hallucinations.

**Fig. 1:**
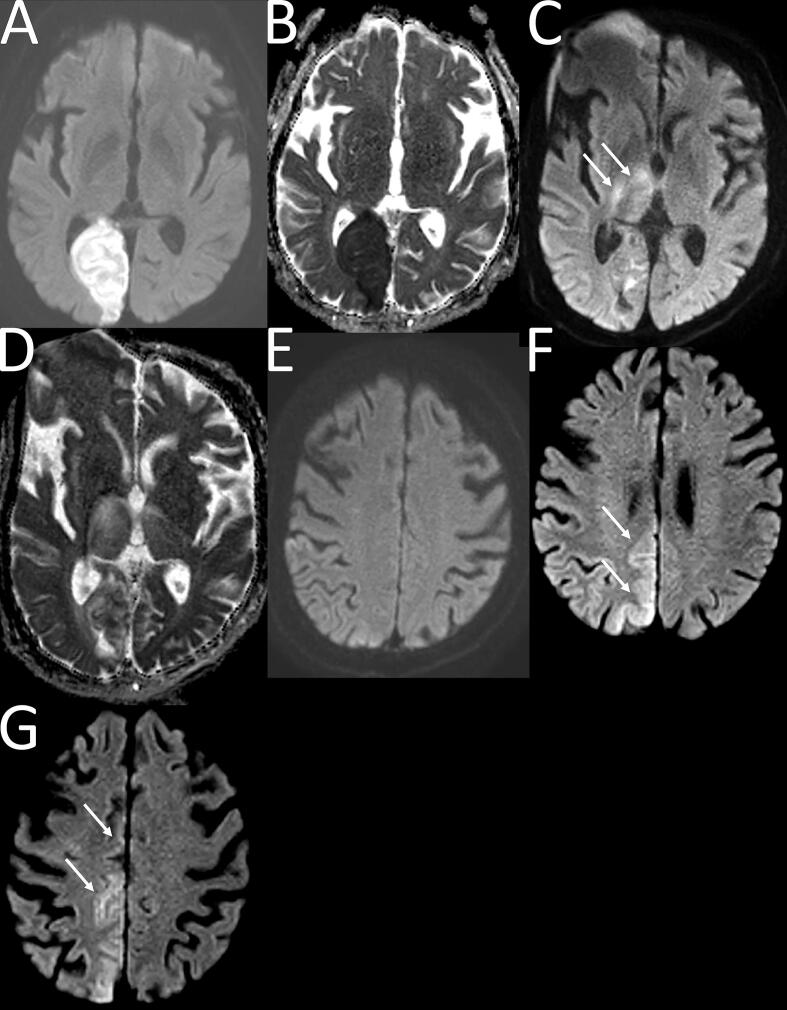
Panel of MRI scans of the brain. **Legend: 1A – 1B** show an acute right occipital lobe infarct. **1A =** MRI Diffusion-weighted b1000 image and **1B** = ADC map. Taken together, they show marked diffusion restriction in the right occipital lobe, in keeping with an acute infarct. **1C – 1E** show MRIs following patient’s admission with confusion and seizures. **1C** = Diffusion-weighted b1000 image and 1D = ADC map. Taken together, they show significant resolution of the acute changes, as compared to in 1A – 1B. Changes in the right thalamus (white arrows), not present previously, are likely due to progression of ischaemia. Distortion in the right frontal lobe is artefactual (due to patient movement). **1E** is a diffusion weighted b1000 image which, at this stage, does not show any change in the right medial parietal cortex or the cingulate gyrus. **1F – 1G** show the MRIs taken a few weeks later. Patient continued to have focal motor seizures. **1F** and **1G** are diffusion-weighted b1000 images showing a degree of cortical based/gyriform/ ‘ribbon-like’ diffusion restriction (ADC map not shown) in the right medial parietal cortex and extending anteriorly along the cingulate gyrus, not present previously (compared to 1E). Excitotoxicity due to seizure-related activity and extension of ischaemia are possible causes. Given the contiguous cortical involvement of both anterior and posterior circulation territories in the context of on-going seizures, seizure-related changes are favoured.

He had a past medical history of an ischaemic stroke affecting the right posterior cerebral artery territory, which had occurred about three months earlier. MRI done at the time demonstrated an acute right occipital lobe infarct (Fig. 1A–B). He also had caecal cancer, diagnosed two years earlier, with metastasis to the liver and lungs, treatment of which had included a right hemicolectomy and palliative chemotherapy. He suffered from type two diabetes mellitus and hypertension.

**Fig. 2:**
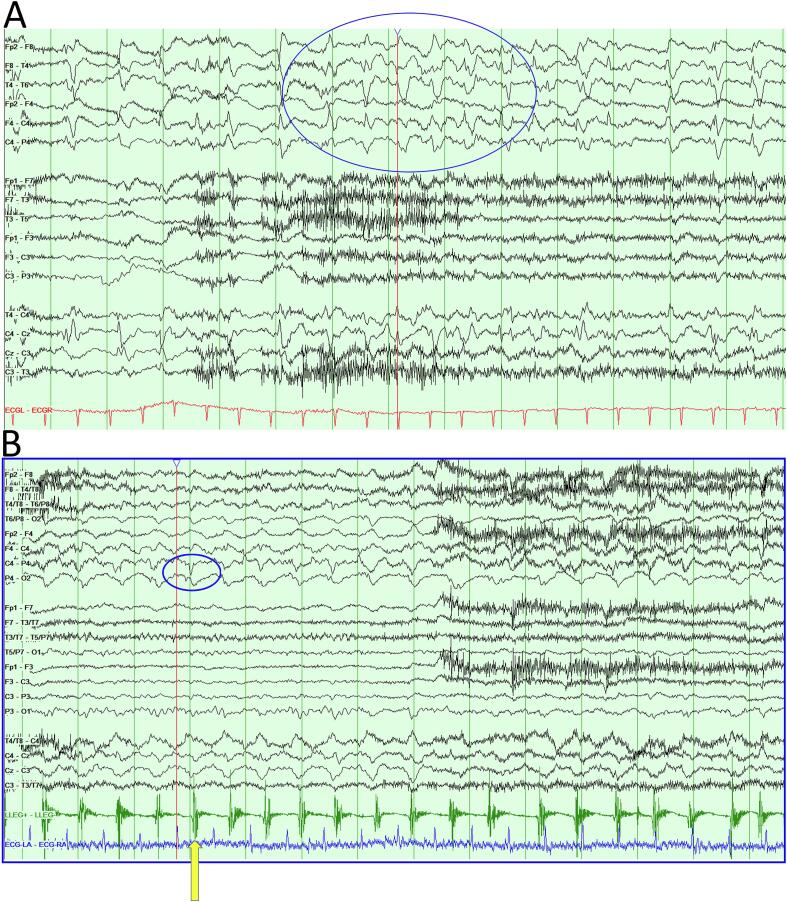
Panel of electroencephalograms (EEGs). **Legend: 2A – 2B** show a bipolar montage of the patient’s EEG. **2A** shows continuous 1–2 Hz sharp waves over the right centro-temporal areas. Some are encompassed in the blue circle for ease of reference. The changes are consistent with focal seizures. **2B** shows synchronous and continuous bursts of raised amplitude sharp waves across the right hemisphere. These are located maximally over the temporal and parietal regions, at a frequency of around 1–2 Hz. They are characteristic of epileptiform discharges. Each is time-locked with an EMG motor discharge (green trace) corresponding to left leg movement. For example, the motor discharge pointed to with the yellow arrow appears just after the epileptiform discharge in the blue circle. Similar is seen elsewhere in the study.

His admission to hospital on this occasion had occurred because he became acutely confused from his normal fully orientated baseline. On admission, his wife reported that he had suddenly become drowsy and his speech did not make sense. He was repeating sentences and had become fixated on particular objects. Within a few days, he became mute and was exhibiting frequent eyelid flickering and rhythmic movements of his mouth. It was suspected that he was having seizures and he was empirically loaded with levetiracetam; a 2,000 mg bolus followed by 1,000 mg twice daily maintenance. CT brain imaging showed a chronic right occipital lobe infarct and old superior bifrontal infarcts. MRI (Fig. 1C–E) showed some maturation of the previous right occipital infarct. There were changes in the right thalamus, not apparent on the previous MRI, deemed to be due to progression of the right posterior circulation ischaemia. There was no evidence of brain metastases from his caecal cancer. Electroencephalogram (EEG) showed continuous 1–2 Hz sharp waves over the right centro-temporal areas (Fig. 2A). Rhythmic movements of the mouth and eyelids were noted on the concurrent video. In the clinical context, the findings were compatible with focal status epilepticus. Advice was given remotely by neurology to increase levetiracetam to 1,500 mg twice daily. There appeared to be clinical resolution with this treatment for a period of days. He then developed continuous involuntary jerking movements of the left leg with retained awareness. The movements continued for a week before inpatient neurology consultation was sought.

On neurological assessment, involuntary jerking movements of the left leg were evident, despite which the patient was alert, orientated, and able to give a history (see Video 1 − https://photos.app.goo.gl/9T4toXbSF8CV1tiEA, also available in Supplementary Data). There was a mild left hemiparesis. Ophthalmological examination showed a complete left homonymous hemianopia (i.e. with no macula sparing) as well as a right upper quadrantanopia; only the right lower quadrant of vision was intact. Visual acuities unaided were 6/9 in the right eye and 6/12 in the left eye. The rest of the neurological and general medical examinations were unremarkable. It was at this point that he volunteered the intermittent distortions of his vision, which he felt had started during this admission, although he was not sure exactly when. The distortions of his vision were present at the time of his neurological assessment, during which the jerking movements of his left leg were ongoing.

A repeat EEG showed synchronous and continuous bursts of raised amplitude sharp waves across the right hemisphere, particularly the temporal and parietal regions, at a frequency of around 1–2 Hz. These were characteristic of epileptiform discharges. The left leg jerks on both clinical observation and EMG recording over the left tibilais anterior were time-locked with the right-sided epileptiform discharges on EEG, with each motor discharge appearing just after a sharp wave (Fig. 2B). The clinico-EMG-EEG correlation was thus compatible with focal status epilepticus, or specifically, epilepsia partialis continua (EPC, a focal motor status epilepticus with typically preserved consciousness) [8]. The patient’s visual and somaesthetic symptoms were diagnosed as AIWS. AIWS symptoms are characteristically intermittent or transient, even with fixed aetiology [6], [9].

A repeat MRI (Fig. 1F–G) showed new cortically based changes in the right medial parietal cortex, extending anteriorly along the cingulate gyrus. Whilst progression of ischaemia was considered as a possible cause of these appearances, given the contiguous cortically based involvement of posterior and anterior circulation territories, in the context of ongoing seizures, seizure-related excitotoxicity was favoured as the cause.

The patient was loaded with phenytoin 1,200 mg, followed by a 100 mg three-times-daily maintenance. His myoclonic jerking resolved soon after this, and the EEG improved to slow activity over the right hemisphere with no epileptiform changes. His AIWS symptoms also resolved within days of commencing this treatment. He was discharged home and passed away peacefully a month later following a general medical decline, during which he was supported by the community palliative care team.

## 3 Discussion

The perceptual distortions reported by our patient were multimodal, both visual and somaesthetic, hence would be characterised as AIWS type C [5]. These distortions were both egocentric, with the perception that certain body parts were larger (macrosomatognosia) or occupied more space (“hyperschematia” in the terminology of Todd, 1955) [6], and allocentric, the perception that items in the environment were larger (macropsia) or smaller (micropsia) along with changed colour perception (dyschromatopsia). These perceptual distortions differed from visual hallucinations in that there was no absence of adequate perceptual stimulus to account for them. Moreover, our patient had preserved insight into the nature of his perceptual experiences.

In this patient, AIWS symptoms were paroxysmal, and did not cause any marked impairment in functioning. As to cause, this was AIWS of central type, with right visual cortex stroke complicated by EPC as the underlying medical condition. Despite his history of caecal cancer, there was no neuroimaging evidence of brain metastases as the cause of his seizures.

Although listed amongst the recognised causes of AIWS, epilepsy may account for only around 3 % of cases [4], [5]. AIWS associated with focal onset epilepsy arising in the right occipital region has been described [10], and also in association with right temporo-occipital non-convulsive status epilepticus associated with cerebral venous sinus thrombosis and COVID-19 infection [11]. However, we have not identified any previous report of AIWS in association with EPC. We postulate that the improvement of AIWS with effective seizure management in this case points to seizures as a compounding or contributory factor to the AIWS symptoms here, with stroke as the underlying structural cause of both. Although the epileptiform discharges in our case did not maximally involve the motor areas, their time-locked correlation with the EMG motor discharges strongly supports their role in explaining the leg movements. This aligns with a recognised margin of error in the somatotopic representation of scalp-recorded maximal voltages, where cortical activity related to motor function may not always localise precisely to expected regions. This is particularly relevant in EPC, where the absence of EEG-detected epileptiform activity in the expected area often suggests that the cortical involvement is too small to record or originates from subcortical structures, as seen in myoclonia continua [12], [13], [14].

A lesion mapping study has suggested that AIWS type B (visual distortions) may generally be associated with right hemisphere visual pathway damage, whereas patients with somaesthetic symptoms (types A and C) had more disparate lesions, albeit always right-sided [15]. AIWS type B accounted for nearly three-quarters of the patients in this report, as in other studies, and most were of vascular aetiology. Nevertheless, similar right hemisphere localisation may be evident in cases of epileptic origin [10], [11], including the current case, suggesting that AIWS related to seizures may be of localising value to the right hemisphere. In addition to pointing to the neuroanatomical substrates of AIWS, this localisation may also aid in understanding the pathogenesis of these perceptual distortions, which may be understood as “pathologies of sensory input”. Involvement of extrastriate visual cortex (Brodmann areas 18 and 19) and adjacent white matter tracts such as the ventral occipital fasciculus, which passes through the temporo-parietal-occipital junction, may result in a failure of visual and somatosensory integration [15]. Sparing of parietal cortex has been suggested to account for the absence of somaesthetic distortions in one AIWS type B patient [11].

## 4 Conclusions

Perceptual distortions, visual (metamorphopsia) and/or somaesthetic, define the AIWS. Perceptual distortions are not hallucinations, since there is adequate perceptual stimulus to account for them. Although most usually migrainous in origin, AIWS may sometimes be due to stroke and/or seizures. AIWS of vascular or epileptic aetiology is most often associated with right hemisphere pathology, especially in the temporo-parietal-occipital region, hence may be of localising value. The pathophysiology of AIWS may be a failure of visual and somatosensory integration. This case captures an unusual association between AIWS and EPC in the context of post-stroke epilepsy. We hope that it will spark further research on the cortical intersection between AIWS, epilepsy, and stroke, an area that remains underexplored in the literature.

## 5 Ethical compliance statement

Written informed consent for publishing was obtained from the patient. We confirm that the approval of an institutional review board was not required for this work. We confirm that we have read the Journal's position on issues involved in ethical publication and affirm that this work is consistent with those guidelines.

## 6 Declaration of Generative AI and AI-assisted technologies in the writing process

During the preparation of this work, the authors did not use generative AI and AI-assisted technologies.

## 7 Funding Sources

G.K.M. is supported by a National Institute for Health and Care Research (NIHR) Clinical Lectureship (CL-2022–07-002), Academy of Medical Sciences (AMS) Starter Grant for Clinical Lecturers (REF: SGL030\1029), and an Epilepsy Research Institute Emerging Leader Fellowship (F2401). For the purpose of open access, the author has applied a Creative Commons Attribution (CC BY) license to any Author Accepted Manuscript version arising.

## 8 Financial disclosures for previous 12 months

G.K.M. discloses i) an Angelini grant to be paid to University of Liverpool as co-applicant on the PROPEL study, which is unrelated to the submitted work; ii) Honoraria to be paid to University of Liverpool for delivering a lecture at an educational event sponsored by Angelini which was unrelated to the submitted work.

## CRediT authorship contribution statement

**Gashirai K. Mbizvo:** Writing – review & editing, Supervision, Funding acquisition, Formal analysis, Data curation, Conceptualization. **Viraj Bharambe:** Writing – review & editing, Formal analysis. **Brython Hywel:** Data curation, Formal analysis, Investigation, Methodology, Writing – review & editing. **Shubhabrata Biswas:** Writing – review & editing, Formal analysis, Data curation. **Andrew J. Larner:** Writing – review & editing, Writing – original draft, Supervision, Conceptualization.

## Declaration of competing interest

The authors declare that they have no known competing financial interests or personal relationships that could have appeared to influence the work reported in this paper.
