# Supplementary material for: Alice in Wonderland Syndrome: Localising insights from right visual cortex stroke complicated by epilepsia partialis continua
Source: Epilepsy Behav Rep. 2025 Jan 29;29:100745. doi: 10.1016/j.ebr.2025.100745 (PMC11840215; doi:10.1016/j.ebr.2025.100745)
Supplement: Supplementary Data 1 [file mmc1.docx]

**Multimedia legends**

**Video 1:** a video of the patient, demonstrating focal jerking movements of the left leg, consistent with myoclonus. The patient retains full awareness and can obey commands. The movements are seen at rest and continue with action (the patient is asked to lift the leg up). Some weakness of the left leg is evident from failure to hold the leg up against gravity continuously. The movements seen and epileptiform discharges later correlated with these movements on electroencephalography are consistent with focal motor status epilepticus (epilepsia partialis continua). The video is available here: <https://photos.app.goo.gl/9T4toXbSF8CV1tiEA>
